# Factors Predict Prolonged Wait Time and Longer Duration of Radiotherapy in Patients with Nasopharyngeal Carcinoma: A Multilevel Analysis

**DOI:** 10.1371/journal.pone.0109930

**Published:** 2014-10-14

**Authors:** Po-Chun Chen, Ching-Chieh Yang, Cheng-Jung Wu, Wen-Shan Liu, Wei-Lun Huang, Ching-Chih Lee

**Affiliations:** 1 Department of Radiation Oncology, Pingtung Christian Hospital, Pingtung, Taiwan; 2 Department of Radiation Oncology, Chi-Mei Medical Center, Tainan, Taiwan; 3 Department of Otolaryngology, Kaohsiung Veterans General Hospital, Kaohsiung, Taiwan; 4 Department of Radiation Oncology, Kaohsiung Veterans General Hospital, Kaohsiung, Taiwan; 5 Department of Otolarygology, Dalin Tzu Chi Hospital, Buddhist Tzu Chi Medical Foundation, Chiayi, Taiwan; 6 Cancer Center, Dalin Tzu Chi Hospital, Buddhist Tzu Chi Medical Foundation, Chiayi, Taiwan; 7 School of Medicine, Tzu Chi University, Hualin, Taiwan; Van Andel Institute, United States of America

## Abstract

**Purpose:**

Radiotherapy with or without chemotherapy is the primary treatment for patients with nasopharyngeal carcinoma (NPC). It wastes time from diagnosis to treatment. Treatment time of radiotherapy generally takes at least seven weeks. The current study aimed to evaluate factors associated with prolonged wait time and longer duration of radiotherapy in NPC patients.

**Methods and Materials:**

From Taiwan's National Health Insurance research database, we identified 3,605 NPC patients treated with radiotherapy between 2008 and 2011. Wait time was calculated from the date of diagnosis to the start of radiotherapy. The impact of each variable on wait time and duration of radiotherapy was examined by multilevel analysis using a random-intercept model.

**Results:**

The mean wait time and duration of radiotherapy were 1.78±3.33 and 9.72±7.27 weeks, respectively. Multilevel analysis revealed prolonged wait time in patients aged 45–65 years, those receiving radiotherapy alone, those with more comorbidities, those with low SES, and those living in eastern Taiwan. A prolonged duration of radiotherapy was associated with receipt of concurrent chemoradiotherapy, more comorbidities, and moderate SES.

**Conclusions:**

Understanding the factors associated with longer wait times and duration of radiotherapy in patients with NPC may help healthcare providers better assist both these patients and potentially those with other head-and-neck cancers.

## Introduction

Nasopharyngeal carcinoma (NPC) is endemic in Southeast Asia, with an annual incidence of 6.17 per 100,000 in Taiwan; its annual incidence in Western countries, by contrast, is <1 per 100,000 [1]. Radiotherapy with or without chemotherpy, which has long been the primary treatment for NPC, varies slightly in treatment modalities [2], [3]. Although NPC is highly radiosensitive, a high failure rate is noted in patients with advanced stage. Treatment stratigies and some time factors, such as wait time or length of treatment, have yet to be optimized [4]–[5].

The impact of time delay on disease control has been investigated in patients with head-and-neck cancers [6]. Moreover, a previous report showed that a treatment delay of>40 days was significantly associated with poorer survival rates in early-stage head-and-neck cancer patients [7]. A longer course of radiotherapy may result in poor disease control in early-stage NPC patients (>12 weeks) or early-stage head-and-neck cancer patients (>7 weeks) [8], [9]. It is important to raise awareness of time delay and prolonged treatment time for decision makers in clinical practice. At present, it remains unclear which factors are associated with time delay and a prolonged duration of radiotherapy in NPC.

We used the nationwide claims data from Taiwan's National Health Insurance (NHI) research database to analyze NPC patients who received radiotherapy between 2008 and 2011. This database provides basic demographic data as well as hospital characteristics, patient characteristics and treatment modality. We sought to identify key factors associated with prolonged wait time and a longer duration of radiotherapy in NPC patients. In terms of improving treatment effects, we hope these information may help to improve future public health stategies and welfare policies.

## Patients and Methods

### Ethical consideration

This study was approved by the Institutional Review Board of Buddhist Dalin Tzu Chi General Hospital, Taiwan. Review board requirements for written informed consent were waived because all personal identifying information was removed from the dataset prior to analysis.

### Study population

We inspected 5,026 NPC patients who received radiotherapy from Taiwan's NHI research database between 2008 to 2011. Taiwan's NHI program covered 99% of the population after 2003, with chart reviews and patient interviews used to verify the accuracy of diagnosis and treatment coding. Patients who received induction or systemic chemotherapy as the initial treatment were excluded except those who received chemotherapy within 14 days prior to radiotherapy. We also excluded patients who were treated for second irradiation. This left 3,605 patients who matched the inclusion criteria for this study. Basic data collected included wait time, duration of radiotherapy, hospital characteristics, gender, age, treatment modality, Charlson Comorbidity Index Score (CCIS), and patient socioeconomic status (SES).

### Treatment modality

Concurrent chemotherapy regimen mostly used in Taiwan is cisplatin 100 mg/m^2^ every 3 weeks for 3 cycles or weekly cisplatin 40 mg/m^2^, followed by adjuvant cisplatin-based chemotherapy (cisplatin 80 mg/m^2^ D1, 5-FU 1000 mg/m^2^ D1-4, repeat cycle every 4 weeks for 1–3 cycles). External beam irradiation of 66–78 Gy was delivered in 33–39 fractions daily using three-dimensional conformal radiotherapy or intensity-modulated radiotherapy.

### Wait time and duration of radiotherapy

Wait time was calculated from the date of diagnosis to the start of radiotherapy. We used the cutpoint of>4 weeks to define prolonged wait time. Duration of radiotherapy was calculated from the start of radiotherapy to the end of radiotherapy. We used the cutpoint of>10 weeks to define longer duration of radiotherapy.

### Other covariates

SES and urbanization of residence were taken from insurance premiums, using income in Taiwan and urbanization variables previously described [10]. Patients were classified into 3 subgroups: high SES (civil servants, full-time or regular paid personnel with a government affiliation or employees of privately owned institutions), moderate SES (self-employed individuals, other employees, and members of the farmers' or fishermen's associations), and low SES (veterans, low-income families, and substitute service draftees). Severity of comorbidity was based on the modified CCIS as recorded before the diagnosis of NPC. The CCIS is a widely accepted scale used for risk adjustment in administrative claims data sets [11]. Different level of hospitals may have inequalities in treatment delay and clinical management during radiotherapy. The hospitals were categorized by hospital teaching level (medical center, regional hospital, or district hospital) or hospital ownership (porfit, non-profit, or public). The geographic regions were recorded as northern, central, southern, and eastern Taiwan.

### Statistical analysis

The key dependent variables of interest were wait time and duration of radiotherapy. The distribution of diseases was described and compared using chi-squared testing. The continuous variables were compared with one-way ANOVA test. Patient characteristics (age, gender, individual SES, CCIS, urbanization and region of patient residence) and hospital characteristics (including ownership and teaching level) were included in the regression model. In this series, the herarchical linear regression method was used due to the potential clustering effect within a hospital. A hospital-level random effect might account for possible correlations between the wait time and duration of radiotherapy within a hospital's panel. A two-tailed value of p<0.05 was considered significant. All statistical operations were performed using SPSS (version 15, SPSS Inc., Chicago, IL).

## Results

A total of 3,605 NPC patients in Taiwan received radiotherapy from 2008 to 2011. Table 1 summarizes the basic demographic characteristics of these patients. In all, 317 patients (8.8%) had wait times greater than 4 weeks. There were 1404 patients (38.9%) who had longer duration of radiotherapy. The mean duration of radiotherapy is 7.68 weeks and 10.16 weeks in patients who received radiotherapy alone and concurrent chemoradiotherapy (CCRT), respectively. Most patients (87.5%) were younger than 65 years. More than half of patients (59.1%) were treated at a medical center. Most, or 2,970 patients (82.4%), received CCRT. Approximately 15.9% of all patients had low SES. Most patients (72%) had lower CCIS. The mean wait time and duration of radiotherapy were 1.78±3.33 and 9.72±7.27 weeks, respectively (Table 2).

**Table 1:** Demographic characteristics for nasopharyngeal cancer patients from 2008 to 2011 (n = 3,605).

Characteristics	Wait time				P value	Duration of radiotherapy				P value
	Less than 4 weeks (n = 3288)		More than 4 weeks (n = 317)			Less than 10 weeks (n = 2201)		More than 10 weeks (n = 1404)		
	No.	%	No.	%		No.	%	No.	%	
Hospital characteristics										
Ownership					0.008					<0.001
For Profit (n = 1,915)	1,770	53.8	145	45.7		1,114	50.6	801	57.1	
Non-profit (n = 563)	498	15.1	65	20.5		396	18.0	167	11.9	
Public (n = 1,127)	1,020	31.1	107	33.8		691	31.4	436	31.1	
Teaching level					0.442					0.006
Medical center (n = 2,332)	2,131	64.8	201	63.4		1,394	63.3	938	66.8	
Regional (n = 1,121)	1,015	30.9	106	33.4		724	32.9	397	28.3	
District (n = 152)	142	4.3	10	3.2		83	3.8	69	4.9	
Gender					0.498					0.151
Male (n = 2,711)	2,472	75.2	239	75.4		1,637	74.4	1,074	76.5	
Female (n = 894)	816	24.8	78	24.6		564	25.6	330	23.5	
Age group					<0.001					0.185
0–44.99 years (n = 1,239)	1,166	35.5	73	23.0		733	33.3	506	36.0	
45–64.99 years (n = 1,916)	1,739	52.9	177	55.8		1,182	53.7	734	52.3	
Older than 65 years (n = 450)	383	11.6	67	21.1		286	13.0	164	11.7	
Treatment					<0.001					<0.001
CCRT† (n = 2,970)	2,789	84.8	181	57.1		1,705	77.5	1,265	90.1	
RT‡ alone (n = 635)	499	15.2	136	42.9		496	22.5	139	9.9	
Charlson Comorbidity Index Score					<0.001					0.053
Lower than mean (n = 2,597)	2,411	73.3	186	58.7		1,611	73.2	986	70.2	
Higher than mean (n = 1,008)	877	26.7	131	41.3		590	26.8	418	29.8	
Socioeconomic status (SES)					0.031					0.050
High SES (n = 1,857)	1,709	52.0	148	46.7		1,169	53.2	688	49.0	
Moderate SES (n = 1,176)	1,073	32.6	103	32.5		690	31.3	486	34.6	
Table 1, continued										

Characteristics	Wait time				P value	Duration of radiotherapy				P value
	Less than 4 weeks (n = 3288)		More than 4 weeks (n = 317)			Less than 10 weeks (n = 2201)		More than 10 weeks (n = 1404)		
	No.	%	No.	%		No.	%	No.	%	
Low SES (n = 572)	506	15.4	66	20.8		342	15.5	230	16.4	
Urbanization					0.045					0.728
Urban (n = 1,149)	1,056	32.1	93	29.3		692	31.4	457	32.5	
Suburban (n = 1,508)	1,386	42.2	122	38.5		922	41.9	586	41.7	
Rural (n = 948)	846	25.7	102	32.2		587	26.7	361	25.8	
Geographic Region					<0.001					0.021
Northern (n = 1,745)	1,745	53.1	133	42.0		1,122	51.0	756	53.8	
Central (n = 502)	502	15.3	53	16.7		370	16.8	185	13.2	
Southern (n = 946)	946	28.8	113	35.6		636	28.9	423	30.1	
Eastern (n = 95)	95	2.9	18	5.7		73	3.3	40	2.8	

† CCRT, Concurrent chemoradiotherapy.

‡ RT, Radiotherapy.

**Table 2:** Distribution of wait time and duration of radiotherapy for nasopharyngeal cancer patients from 2008 to 2011 by univariate analysis (n = 3,605).

Characteristics	Wait time			Duration of radiotherapy		
	Mean	± SD	P value	Mean	± SD	P value
	1.78	3.33		9.72	7.27	
Hospital characteristics						
Ownership			0.092			0.064
Profit organization (n = 1,915)	1.71	3.45		9.92	7.88	
Non-profit organization (n = 563)	2.06	3.44		9.10	6.19	
Public (n = 1,127)	1.77	3.05		9.69	6.65	
Teaching level			0.226			0.171
Medical center (n = 2,332)	1.72	3.21		9.81	7.34	
Regional (n = 1,121)	1.93	3.54		9.43	7.21	
District (n = 152)	1.77	3.47		10.43	6.55	
Gender			0.803			0.811
Male (n = 2,711)	1.79	3.35		9.70	7.32	
Female (n = 894)	1.76	3.27		9.77	7.11	
Age group			<0.001			0.407
0–44.99 years (n = 1,239)	1.44	2.71		9.90	6.83	
45–64.99 years (n = 1,916)	1.90	3.54		9.68	6.98	
Older than 65 years (n = 450)	2.23	3.85		9.39	9.37	
Treatment			<0.001			<0.001
CCRT† (n = 2,970)	1.47	2.60		10.16	7.49	
RT‡ alone (n = 635)	3.27	5.36		7.68	5.71	
Charlson Comorbidity Index Score			<0.001			<0.001
Lower than mean (n = 2,597)	1.58	2.80		9.37	6.50	
Higher than mean (n = 1,008)	2.30	4.37		10.62	8.90	
Socioeconomic status (SES)			0.008			0.036
High SES (n = 1,857)	1.66	2.99		9.42	6.37	
Moderate SES (n = 1,176)	1.81	3.42		10.04	8.37	
Low SES (n = 572)	2.15	4.08		10.04	7.53	
Table 2, continued						

Characteristics	Wait time			Duration of radiotherapy		
	Mean	±SD	P value	Mean	±SD	P value
Urbanization			0.348			0.482
Urban (n = 1,149)	1.73	3.30		9.61	7.53	
Suburban (n = 1,508)	1.74	3.29		9.89	7.53	
Rural (n = 948)	1.92	3.42		9.58	6.45	
Geographic Region			<0.001			0.698
Northern (n = 1,745)	1.58	3.06		9.68	7.28	
Central (n = 502)	1.87	3.22		9.50	6.40	
Southern (n = 946)	1.99	3.54		9.92	7.81	
Eastern (n = 95)	2.76	5.32		9.55	5.78	

† CCRT, Concurrent chemoradiotherapy.

‡ RT, Radiotherapy.

SD, standard deviation;

### Wait time

Univariate analysis revealed that wait times were prolonged in patients older than 45 years, those who received radiotherapy alone, those with higher CCIS, those with low to moderate SES, and those who did not live in northern Taiwan.

After adjusting for patient and hospital characteristics, the hierachical linear regression revealed significant factors assoicated with wait time as the followings: for those age 45–65 years was 0.25 week longer than those age less than 45 years (p = 0.03); for those with RT alone was 1.78 week longer than those with CCRT (p<0.001); for those with higher morbidities was 0.72 week longer than those with lower comorbidities (p<0.001); for those with low SES was 0.34 week longer than those with high SES (p = 0.029) and those in eastern area was 1.29 week longer than the northern area (Table 3).

**Table 3:** Distribution of wait time and duration of radiotherapy for nasopharyngeal cancer patients from 2008 to 2011 by multivariate analysis using a random-intercept model (n = 3605).

Characteristics	Wait time			Duration of radiotherapy		
	Estimate	95% CI*	p value	Estimate	95% CI*	p value
Intercept	0.97	(0.53–1.41)	<0.001	9.74	(8.77–10.71)	<0.001
Hospital characteristics						
Ownership						
Profit organization	Reference			Reference		
Non-profit organization	0.13	(−0.41–0.68)	0.628	−0.27	(−1.48–0.93)	0.648
Public	0.09	(−0.37–0.55)	0.690	−0.08	(−1.10–0.93)	0.865
Teaching level						
Medical center	Reference			Reference		
Regional	0.20	(−0.21–0.62)	0.336	−0.33	(−1.25–0.59)	0.475
District	−0.07	(−0.74–0.60)	0.831	0.81	(−0.68–2.30)	0.285
Gender						
Male	Reference			Reference		
Female	−0.030	(−0.27–0.21)	0.806	0.08	(−0.45–0.62)	0.312
Age group						
0–44.99 years	Reference			Reference		
45–64.99 years	0.25	(0.02–0.49)	0.030	−0.23	(−0.75–0.28)	0.377
Older than 65 years	−0.03	(−0.41–0.33)	0.840	−0.20	(−1.04–0.62)	0.622
Treatment						
CCRT†	Reference			Reference		
RT‡ alone	1.78	(1.49–2.07)	<0.001	−2.42	(−3.06–−1.77)	<0.001
Charlson Comorbidity Index Score						
Lower than mean	Reference			Reference		
Higher than mean	0.72	(0.48–0.96)	<0.001	1.08	(0.56–1.61)	<0.001
Socioeconomic status (SES)						
High SES	Reference			Reference		
Low SES	0.34	(0.03–0.64)	0.029	0.65	(−0.02–1.33)	0.059
Moderate SES	−0.01	(−0.26–0.23)	0.920	0.65	(0.09–1.20)	0.021
Table 3, continued						

Characteristics	Wait time			Duration of radiotherapy		
	Estimate	95% CI*	p value	Estimate	95% CI*	p value
Urbanization						
Urban	Reference			Reference		
Suburban	−0.18	(−0.45–0.07)	0.159	0.31	(−0.27–0.90)	0.291
Rural	−0.28	(−0.61–0.03)	0.081	−0.09	(−0.81–0.62)	0.796
Geographic Region						
Northern	Reference			Reference		
Central	0.17	(−0.25–0.60)	0.426	0.01	(−0.94–0.95)	0.992
Southern	0.29	(−0.06–0.66)	0.111	0.04	(−0.76–0.85)	0.909
Eastern	1.29	(0.51–2.08)	0.01	−0.21	(−1.94–1.51)	0.805

† CCRT, Concurrent chemoradiotherapy.

‡ RT, Radiotherapy.

CI, confidence interval.

### Duration of radiotherapy

Univariate analysis revealed a longer duration of radiotherapy in patients who received CCRT, with a mean of 10.16 weeks; in those with higher CCIS, with a mean of 10.62 weeks; and in those with low or moderate SES, with a mean of 10.04 weeks.

After adjusting for patient and hospital characteristics, the hierachical linear regression revealed significant factors assoicated with duration of radiotherapy as the followings: for those with RT alone was 2.42 week shorter than those with CCRT (p<0.001); for those with higher morbidities was 1.08 week longer than those with lower comorbidities (p<0.001); for those with moderate SES was 0.65 week longer than those with high SES (p = 0.021) (Table 3).

## Discussion

Our study demonstrated that higher CCIS was an independent factor for both prolonged wait time and longer duration of radiotherapy in NPC patients. Lower SES was an independent factor for time delay but not for duration of radiotherapy. CCRT was associated with the greatest duration of radiotherapy, prolonging treatment 2.42 weeks more than radiotherapy alone.

The strengths of our study include the endemic nature of NPC in Taiwan, allowing for the collection of a large sample size to make valid estimates and compare treatment modalities. Moreover, the NHI research database captures complete follow-up information, provides comprehensive health care benefits with a moderate cost sharing, and records all treatments. Ongoing validation of the NHI research database is conducted via comparison of chart-based and claims-based records [12]. To avoid causes of delay not identified in our study, we excluded patients with an interval of more than 120 days between diagnosis and start of radiotherapy. To our knowledge, this is the first study investigating the association between time factors and hospital characteristics, patient characteristics, and treatment modality in NPC patients.

As cancer incidence has increased in various parts of the world, so has the demand for radiotherapy for each type and stage of cancer [13], [14]. Radiotherapy facilities are available worldwide, but are often inadequate to the population demands placed on them. In Taiwan, the nearly 60 radiotherapy facilities provide medical care for more than 20 million people. Taiwan's NHI program has provided for the medical needs of Taiwan for 20 years. Nevertheless, treatment delays are common. Similarly, Round et al. [13] compared predictive models for radiotherapy demand. The Methus model estimated a 13.1% increase in need for radiotherapy between 2011 and 2016. In general, treatment delays may result from health policy, patients themselves, or hospital characteristics. In our study, we did not find any significant difference in wait time between medical centers and other types of hospitals. Furthermore, alternating radiotherapy helps to relieve the burden on the system and shorten the wait time. However, such treatment is not indicated for certain cancers.

A literature review reported a negative impact of comorbidity on incidence of treatment complication, quality of life, increased cost of treatment and survival [15]. Assessment of comorbid diseases should be an important part in clinical practice. Moreover, the impact of comorbid diseases on therapeutic decision-making in head and neck cancer has been reported [16]. Comorbidity was assessed with Adult Comorbidity Evaluation (ACE-27) and Charlson Comorbidity Index (CCI). Results showed moderate to strong positive correlation between comorbidity and change in therapeutic decision-making. In our study, higher CCIS is an independent factor for both prolonged wait time and longer duration of radiotherapy in NPC patients. It is important to correct any underlying comorbid diseases prior to and during radiotherapy. Moreover, radiotherapy is a local treatment. The most common treatment-related side effects which lead to unplanned treatment interruptions are severe mucositis and skin reaction. The recovery time depends on the degree of the injury. Some comorbid conditions are associated with delayed wound healing, especially poor nutritional status, vascular disease, and diabetes mellitus [17]. Since the exact cause of treatment interruptions in our study is unknown, possible causes have been discussed using Charlson Comorbidity Index Score instead of a specific comorbid disease. A recently published study has developed a revised comorbidity index for head and neck cancer patients [18]. It is worth investigating this revised comorbidity index in NPC patients in future studies.

In fact, patients with low SES have inequalities in health. They have delays in diagnosis, are offered different treatment modalities than those with higher income, and experience inferior outcomes to those of patients with higher SES, mostly shown in research on breast cancer [10], [12], [19]. A systemic review shows that patients from lower social classes receive significantly less positive socio-emotional utterances, a more directive and a less participatory consulting style, characterized by by significantly less information giving, less directions and less socio-emotional and partnership building utterances from their doctor [20]. In Taiwan, SES does not affect the medical care patients receive, as all receive universal health insurance which reimburses hospitals directly for care. Even so, patients in our study who had low SES had significantly prolonged wait times over others. Thorough communication between doctors and patients is crucial so that mutual understanding can be achieved to improve patients' compliance, thereby reduce prolonged wait times, especially in low SES patients.

An early report from Hong Kong confirmed that interruptions in and prolongation of treatment adversely affect outcomes in radiotherapy for NPC [21]. Other studies have also demonstrated the impact of a longer duration of radiation treatment on local failure risk and overall survival in patients with NPC and other types of head-and-neck cancers [8], [9], [22]. However, there is little evidence to suggest which factors are associated with prolonged duration of radiotherapy. To find a possible correlation, we looked in this study for factors associated with prolonged radiation treatment time. CCRT was associated with the greatest duration of radiotherapy in this study. In general, acute toxicity caused by radiation and chemotherapy is responsible for this. Concurrent chemotherapy would increase acute toxicity over that of radiotherapy alone. Supportive medications to improve symptoms such as odynophagia and severe skin reaction should be provided as early as possible. Kim et al. [23] reported a prescription of a 3-week cycle of 100 mg/m^2^ cisplatin prolonged treatment 1.8 weeks more than weekly cisplatin 30 mg/m^2^. Current evidence suggested no difference in survival between the two chemotherapy groups. In our study, concurrent chemotherapy regimen mostly used is either cisplatin 100 mg/m^2^ every 3 weeks for 3 cycles or weekly cisplatin 40 mg/m^2^. Weekly Cisplatin that causes less complications may be effectively used to avoid treatment interruptions, thereby shorthen the radiation treatment period.

This study has three potential limitations. Firstly, cancer stage was not obtained. However, we excluded patients who had potentially distant metastases by capturing information on the interval between initial chemotherapy and radiotherapy. In fact, the association between cancer stage and time factos has not yet to be identified from previous literatures. Secondly, the diagnosis of NPC and the record of comorbid conditions are dependent on ICD codes. Different coding quality between different levels of hospitals may result in bias. Finally, the assoicaiton of time factors ad NPC outcomes were not explored in this series, and we will lanuch a new study in the furure. However, the NHI program in Taiwan reviews selected charts to verify the accuracy of diagnosis and treatment coding.

## Conclusion

Radiotherapy is a multi-step, time-consuming treatment. It is difficult to determine whether the time delay related to health policy, patient factors, hospital characteristics, or some combination of these. With available administrative data, we found significant factors associated with prolonged wait time and longer duration of radiotherapy in patients with NPC. Our study may help healthcare providers and those responsible for health policy better understand this patient population and even apply these results to those with other head-and-neck cancers so as to make informed decisions on how to reduce wait time and length of treatment in the future. The impact of both wait time and duration of radiotherapy on survival remains to be investigated.
